# Neural Representations of Emotions in Visual, Auditory, and Modality‐Independent Regions Reflect Idiosyncratic Conceptual Knowledge

**DOI:** 10.1002/hbm.70040

**Published:** 2024-10-12

**Authors:** Chuanji Gao, Sewon Oh, Xuan Yang, Jacob M. Stanley, Svetlana V. Shinkareva

**Affiliations:** ^1^ School of Psychology Nanjing Normal University Nanjing China; ^2^ Department of Psychology, Institute for Mind and Brain University of South Carolina Columbia South Carolina USA

**Keywords:** conceptual knowledge, emotion, facial expressions, modality‐independent, vocal expressions

## Abstract

Growing evidence suggests that conceptual knowledge influences emotion perception, yet the neural mechanisms underlying this effect are not fully understood. Recent studies have shown that brain representations of facial emotion categories in visual‐perceptual areas are predicted by conceptual knowledge, but it remains to be seen if auditory regions are similarly affected. Moreover, it is not fully clear whether these conceptual influences operate at a modality‐independent level. To address these questions, we conducted a functional magnetic resonance imaging study presenting participants with both facial and vocal emotional stimuli. This dual‐modality approach allowed us to investigate effects on both modality‐specific and modality‐independent brain regions. Using univariate and representational similarity analyses, we found that brain representations in both visual (middle and lateral occipital cortices) and auditory (superior temporal gyrus) regions were predicted by conceptual understanding of emotions for faces and voices, respectively. Additionally, we discovered that conceptual knowledge also influenced supra‐modal representations in the superior temporal sulcus. Dynamic causal modeling revealed a brain network showing both bottom‐up and top‐down flows, suggesting a complex interplay of modality‐specific and modality‐independent regions in emotional processing. These findings collectively indicate that the neural representations of emotions in both sensory‐perceptual and modality‐independent regions are likely shaped by each individual's conceptual knowledge.

## Graphical abstract

We found that brain representations of emotions in both visual and auditory cortices, as well as in modality‐independent regions, are shaped by the conceptual understanding of emotions. These modality‐specific and modality‐independent brain regions are interconnected through both bottom‐up and top‐down information flows.

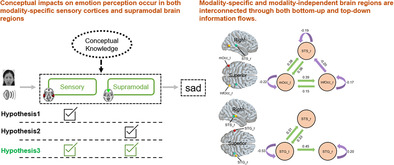

**Summary**

- Brain representations of emotions in visual and auditory cortices are shaped by conceptual understanding of emotions.
- Conceptual impacts on emotion perception also occur at a modality‐independent level in the superior temporal sulcus.
- Modality‐specific and modality‐independent brain regions are interconnected through both bottom‐up and top‐down information flows.

## 1 Introduction

Perception of emotions is a crucial ability in humans. The success of social interactions often hinges on accurately perceived emotions, and dysfunctions in this ability are associated with various psychological disorders such as alexithymia, social anxiety, and autism. A key question in cognitive science is how humans decode emotions through signals from multiple modalities, including facial and vocal expressions. One possibility is that emotion perception is a bottom‐up, direct read‐out of stimulus cues (Ekman and Cordaro 2011), without involving conceptual knowledge of expressions, subjective experiences, bodily responses, typical situations, and action tendencies associated with specific emotions (see Barrett and Lindquist 2008; Niedenthal 2008 for a discussion on conceptual knowledge).

However, recent theories suggest that emotion perception also involves the integration of conceptual knowledge into the perceptual process (Barrett 2014; Freeman and Ambady 2011; Freeman, Stolier, and Brooks 2020; Lindquist 2017; Lindquist, Satpute, and Gendron 2015). For instance, according to the constructionist view, emotion concepts are essential in shaping how we perceive and interpret emotions. These concepts, conveyed through language, help organize the diverse and variable sensory inputs into coherent emotion categories, thereby influencing our perception of emotional stimuli (Lindquist 2017; Lindquist and Gendron 2013; Lindquist, Satpute, and Gendron 2015). Though there is growing evidence that conceptual knowledge influences emotion perception (Brooks and Freeman 2018; Gendron et al. 2012; Lindquist et al. 2006; Nook, Lindquist, and Zaki 2015), the neural basis of this conceptual impact remains unclear.

A recent study examined the neural basis of conceptual effects on facial emotion perception using functional magnetic resonance imaging (fMRI) (Brooks et al. 2019). The study quantified the conceptual similarity between pairs of six emotions: anger, disgust, fear, happiness, sadness, and surprise. Representational similarity analysis (RSA) (Kriegeskorte, Mur, and Bandettini 2008) revealed that each participant's conceptual structure significantly predicted the neural representational structure in the right fusiform gyrus (Brooks et al. 2019). This finding suggests that brain representations of emotion categories in visual‐perceptual regions may be shaped by conceptual understanding of emotions.

Brooks et al. (2019) demonstrated that conceptual influences on emotion perception occur at the perceptual processing level, yet the underlying mechanisms are not fully understood. First, it is uncertain whether conceptual knowledge shapes the brain's representations of emotion categories within auditory regions. Although previous studies have identified perceptual regions that process emotional content in facial and vocal expressions (Brück, Kreifelts, and Wildgruber 2011; Kragel et al. 2019; Sabatinelli et al. 2011), the extent to which conceptual knowledge modifies these representations, particularly in auditory areas, remains ambiguous. According to one theory, voice is not simply an “auditory face,” and auditory processing may not mirror visual mechanisms (Schirmer 2018; Schirmer and Adolphs 2017). From this perspective, the conceptual impacts on the brain's representations of facial emotions in visual regions may not necessarily apply to vocal emotions in auditory regions. Conversely, it has been proposed that the perception of faces and voices involves notably similar cognitive and neural mechanisms (Belin 2017; Belin, Fecteau, and Bedard 2004; Yovel and Belin 2013). Given this framework, it is plausible to expect that conceptual knowledge might influence the brain's representations of emotions in auditory regions for voices in a manner similar to its effects on faces in visual regions.

Whether conceptual impacts on emotion perception occur at a modality‐independent level or not is also unclear. Research indicates the presence of a supra‐modal neural system that encodes emotions, independent of visual and auditory modalities, encompassing brain regions like the superior temporal sulcus (STS) and the medial prefrontal cortex (Gao and Shinkareva 2021; Gao, Weber, and Shinkareva 2019; Kim, Shinkareva, and Wedell 2017; Lettieri et al. 2024; Peelen, Atkinson, and Vuilleumier 2010; Shinkareva, Gao, and Wedell 2020; Young, Frühholz, and Schweinberger 2020). However, these studies did not investigate the fundamental impact that conceptual knowledge could have on the supra‐modal brain representations of emotions. It remains uncertain whether this effect occurs in a sensory‐dependent manner, a modality‐independent manner, or both.

To investigate the neural underpinnings of conceptual influences on emotion perception, we conducted two experiments. In Experiment 1, we measured each participant's idiosyncratic conceptual similarity between each pair of seven emotions (six basic emotions and neutral), similar to a prior study (Brooks et al. 2019). The objective of Experiment 1 was to explore the variability in emotional conceptual knowledge among people and to assess its low‐dimensional representations and validity. Experiment 2 presented participants with both facial and vocal emotional stimuli while recording their fMRI responses. This dual‐modality approach enabled us to examine both modality‐specific and modality‐independent effects on emotion perception. We conducted univariate activation analyses to pinpoint the modality‐specific and modality‐independent brain regions and utilized RSA to delve deeper into how conceptual knowledge influences brain representations in both sensory‐perceptual and modality‐independent areas. Additionally, we employed dynamic causal modeling to determine the direction of information flow between brain regions implicated in the effects of conceptual knowledge on emotion perception.

## 2 Method

### 2.1 Participants

The study involved 132 participants from the University of South Carolina community across the two experiments. A total of 103 volunteers (12 males, 79 females, and 12 individuals did not report their sex; mean age = 20.2 ± 1.5 years) participated in Experiment 1. A total of 29 volunteers (13 males, mean age = 21.0 ± 2.0 years) participated in Experiment 2. All participants provided written consent according to the University of South Carolina Institutional Review Board's approved procedures. Experiment 1 participants received course credit, whereas Experiment 2 participants were paid for their participation. In Experiment 1, two participants were disqualified for repetitive responses across all trials, and 11 were excluded due to technical issues with data recording, resulting in a final sample of 90 participants (11 males, mean age = 20.3 ± 1.5 years). In Experiment 2, all but two participants were right‐handed, native English speakers. One individual, although not a native English speaker, was raised in an English‐speaking country, and another was left‐handed. Data from four participants were discarded due to data collection artifacts and technical problems during auditory or visual stimulus presentation, leaving 25 participants (11 males, mean age = 20.9 ± 1.7 years) in the final sample. The sample size for each experiment was not predetermined with power analyses; however, the sample sizes utilized in this study are consistent with those employed in similar research (Brooks et al. 2019). All participants had normal or corrected‐to‐normal vision and reported no hearing issues, neurological disorders, or psychiatric conditions.

## 3 Experiment 1: Conceptual Similarity Behavioral Rating

### 3.1 Stimuli

The stimuli consisted of 40 words and phrases selected for the conceptual similarity behavioral rating task. These words were sourced from a prior study where participants were instructed to list features of category exemplars (Brooks and Freeman 2018). In this previous study, participants were prompted to “list the top 5 bodily feelings, thoughts, or actions” they associated with each of six emotion categories: angry, disgusted, fearful, happy, sad, and surprised. The 40 most frequently occurring words and phrases across all emotions and participants, independent of the specific emotion category that prompted them, were then compiled. These selected words are listed in Table S1.

### 3.2 Procedure

The study consisted of seven blocks, one for each of the seven emotion categories: angry, disgusted, fearful, happy, sad, surprised, and neutral. We included “neutral” as a pivot category on top of the six basic emotions (van Rijn and Larrouy‐Maestri 2023). Following Brooks and Freeman (2018), within each block, participants evaluated the 40 word and phrase stimuli based on their relevance to the respective emotion category using a 7‐point scale (Brooks and Freeman 2018). For instance, participants were asked, “On a scale from 1 (*not at all*) to 7 (*extremely*), how related is ‘yelling’ to the emotion Anger?” This resulted in a total of 280 trials (7 emotions × 40 words).

### 3.3 Data Analyses

#### 3.3.1 Distributions of Conceptual Dissimilarity Across Participants

To visualize the inter‐subject variability in conceptual knowledge, we plotted the distributions of conceptual dissimilarity across participants for each pair of emotions. We defined the conceptual dissimilarity between a pair of emotions as the Pearson correlation distance between two vectors representing the ratings given to each emotion category in relation to 40 word and phrase stimuli. The choice of Pearson correlation distance was motivated by its equivalence to the squared Euclidean distances of normalized pattern vectors, coupled with its straightforward interpretability, following a prior study (Brooks et al. 2019). Dissimilarity was calculated by subtracting the Pearson correlation coefficients from 1. Consequently, a dissimilarity value of 0 indicates perfect conceptual similarity between two emotions, whereas a higher value suggests a greater conceptual dissimilarity.

#### 3.3.2 MDS and Hierarchical Clustering Analysis

To explore a lower‐dimensional representation of the seven emotions in conceptual knowledge, we employed Kruskal's nonmetric multidimensional scaling (MDS), using the classical solution from metric MDS as the initial configuration (Cox and Cox 2000; Shinkareva, Wang, and Wedell 2013). The input data for the MDS analyses consisted of dissimilarities calculated using Pearson correlation distances. We aimed for a two‐dimensional solution, and the iterative algorithm achieved convergence after 20 iterations. To further visualize the clustering of emotion categories based on conceptual knowledge, we also performed a hierarchical clustering analysis using Ward's criterion (Murtagh and Legendre 2014).

## 4 Experiment 2: fMRI

### 4.1 Stimuli

The stimuli consisted of videos displaying emotional facial expressions and audio clips portraying emotional voices, sourced from the Ryerson Audio‐Visual Database of Emotional Speech and Song (Livingstone and Russo 2018). For each of the seven emotional categories—anger, disgust, fear, happiness, sadness, surprise, and neutral—there were 32 faces and 32 voices, featuring 8 male and 8 female actors. These actors expressed two neutral statements, either “Kids are talking by the door” or “Dogs are sitting by the door,” resulting in 16 face and 16 voice stimuli for each statement. All original face and voice stimuli were edited to a duration of 3 s, with start times identified using FFmpeg (https://ffmpeg.org/). Voice intensity levels were standardized to a standard intensity level 70 SPL (dB) using Praat software (Boersma and Weenink 2009). Additionally, stimuli from four additional actors were employed for catch trials (see section *fMRI task* for details), involving the actors articulating the statements “Kids are talking by the door” and “Dogs are sitting by the door.” For practice trials, faces and voices from an additional set of four new actors were used.

### 4.2 Procedure

#### 4.2.1 Overview

**FIGURE 1:**
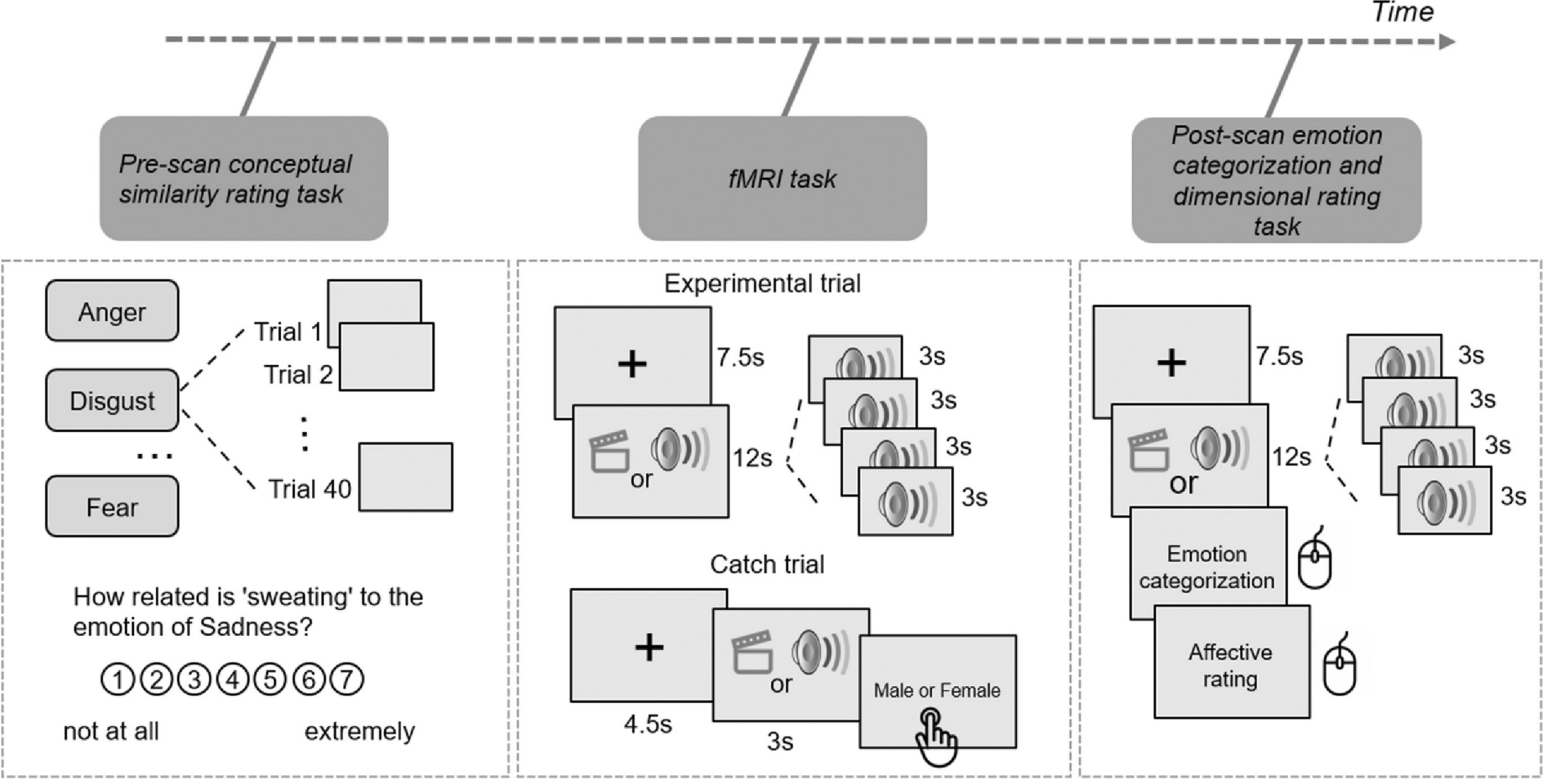
Overview of Experiment 2. Participants performed a pre‐scan conceptual similarity rating behavioral task, completed a 1‐h fMRI scan, and concluded with a post‐scan emotion categorization and dimensional rating behavioral tasks.

Participants performed a pre‐scan conceptual similarity rating behavioral task, completed a 1‐h fMRI scan, and concluded with a post‐scan behavioral task. After finishing the pre‐scan task in a behavioral room, participants underwent a final MRI safety screening before entering the scanning room. Inside the scanner, participants were presented with facial and vocal expressions and carried out a task. Subsequently, the participants completed a post‐scan emotion categorization task in a behavioral room (Figure 1).

#### 4.2.2 Pre‐Scan Conceptual Similarity Rating Task

The pre‐scan task mirrored Experiment 1, wherein participants assessed the relevance of 40 word and phrase stimuli to specific emotion categories using a 7‐point scale. The task comprised 280 trials in total, multiplying the 40 stimuli by 7 emotion blocks (angry, disgusted, fearful, happy, sad, surprised, and neutral). An example question posed was “How related is ‘sweating’ to the emotion of Sadness?” The questions were organized by emotion, with both the emotion blocks and the corresponding words within each block presented in a random order.

#### 4.2.3 fMRI Task

The scanner task was divided into four sessions: two presented facial expressions, and the other two presented vocal expressions. Each session featured four blocks, with two blocks dedicated to actors conveying “Kids are talking by the door” and the remaining two to “Dogs are sitting by the door.”. Each block contained seven trials, showcasing one of the seven emotions through the facial or vocal expressions of four actors, matched by sex and emotion for each trial. Trials commenced with a 4.5‐s fixation cross, followed by the presentation of four actors' faces or voices for 3 s each, and concluded with a 3‐s fixation cross. Presenting stimuli of the same emotion from four different actors sequentially helped maintain a consistent emotional state in participants, leading to more stable brain activity patterns. This also enhanced the signal‐to‐noise ratio by averaging brain responses across trials, thereby reducing random noise. This approach is especially important for dynamic causal modeling (DCM), where accurate estimation of connectivity patterns relies on clear and consistent signals. Blocks were separated by a 12‐s rest period. To sustain attention, two catch trials were interspersed at the end of a block within each session, asking participants to determine the actor's sex, starting with a 4.5‐s fixation cross, a single 3‐s face or voice presentation, and ending with a “Male or Female” prompt. Responses were to be made swiftly and accurately with a response glove, using the index finger for “male” and the middle finger for “female.” The stimuli varied between the two facial and two vocal sessions, with each session featuring a unique set of actors. For instance, Session 1 might have featured Actors 1 to 8, while Session 2 featured Actors 9 to 16. Actor combinations also varied across blocks, ensuring a diverse representation across trials. Consequently, each session comprised 114 trials, combining 7 emotions across 4 actors with 2 additional catch trials. Sessions were arranged in a counterbalanced order across participants, alternating between facial and vocal sessions. The sequence of blocks and the order of the seven trials within each block were randomized to enhance task variability.

#### 4.2.4 Post‐Scan Emotion Categorization and Dimensional Rating Task

In the post‐scan behavioral task participants were exposed to the same stimuli as in the fMRI experiment, but with a distinct task. Unlike the in‐scanner task, the post‐scan task involved emotion categorization and valence‐arousal assessment. Each trial commenced with a 1‐s fixation cross, succeeded by a sequence of four faces or voices, each lasting 3 s. Subsequently, an emotion categorization prompt appeared, prompting participants to identify the most fitting emotion from a selection of seven. Following this, we presented a valence‐arousal rating grid with the horizontal axis reflecting valence, varying from negative to positive, and the vertical axis reflecting arousal, varying from low to high, enabling participants to evaluate the emotion's positivity or negativity and its arousal level. Participants selected their responses and performed ratings using a computer mouse.

#### 4.2.5 MRI Acquisition

Imaging data was acquired using a 3‐T Siemens MAGNETOM Prisma fit MRI scanner (Siemens, Erlangen, Germany), equipped with a 20‐channel head coil at the McCausland Center for Brain Imaging, University of South Carolina. Whole‐brain functional images were captured using T2*‐weighted echo‐planar imaging (EPI), designed to be sensitive to the blood‐oxygen‐level‐dependent (BOLD) signal contrast. Initially, functional images for the first four participants were obtained with 36 slices, employing the following parameters: slice thickness = 3 mm, repetition time (TR) = 1500 ms, echo time (TE) = 30 ms, flip angle = 62°, field of view (FOV) = 210 mm, in‐plane resolution = 70 pixels, voxel size = 3.3 × 3.3 × 3 mm^3^, using an ascending interleaved acquisition order. Following a pause in data collection due to COVID‐19, the imaging protocol was adjusted. For one participant, functional images were acquired with 48 slices, while for the remaining 24 participants, images were obtained with 50 slices, maintaining the same imaging parameters but adding a GRAPPA multiband acceleration factor of 2. In all cases, a 2‐min fieldmap scan was acquired after the first session to aid in data processing and analysis. High‐resolution anatomical images were obtained after fMRI acquisition through a T1‐weighted (T1w) sequence, with the following parameters: TR = 2530 ms, TE = 1440 ms, flip angle = 7°, FOV = 256 mm, in‐plane resolution = 256 pixels, and voxel size = 1 × 1 × 1 mm^3^.

### 4.3 Data Analyses

#### 4.3.1 Behavioral Analyses

##### 4.3.1.1 Pre‐Scan Ratings

For the pre‐scan conceptual similarity rating data, we calculated the conceptual dissimilarity between pairs of emotions using the Pearson correlation distance. This was done by comparing two vectors, each representing the ratings assigned to each emotion category based on responses to 40 words and phrases, consistent with the methodology of Experiment 1. To investigate a lower‐dimensional representation of the seven emotions within conceptual knowledge and to compare this representation with explicit valence and arousal ratings obtained in the post‐scan task, we applied Kruskal's nonmetric MDS to the data, following the same procedure as in Experiment 1.

##### 4.3.1.2 Correlation Between Conceptual Dissimilarity and Emotion Categorization Performances

To evaluate the association between conceptual knowledge and emotion categorization, we conducted a correlation analysis between the pre‐scan average conceptual dissimilarity matrix and the post‐scan emotion categorization confusion matrix. Given the high accuracy rates, we did not analyze the post‐scan emotion categorization confusion matrix on an individual basis but instead used the overall performance across all participants. Similarly, we used the average conceptual dissimilarity matrix across all participants. Our analysis focused on the group‐specific relationship between conceptual knowledge and emotion categorization rather than on individual‐level analysis.

We computed an overall emotion categorization confusion matrix summarizing the responses from all participants, with rows indicating the correct category and columns reflecting the participants' responses. To transform the confusion matrix into a dissimilarity matrix, we subtracted its off‐diagonal elements from the highest off‐diagonal value. This adjustment reversed the scale, ensuring that larger values in the dissimilarity matrix indicate greater confusion. Next, we extracted the upper triangular values from both the conceptual dissimilarity and the emotion categorization confusion matrices. We then performed a Pearson correlation analysis between the *z*‐scores of these upper triangular values to assess the relationship between conceptual knowledge and emotion categorization.

#### 4.3.2 fMRI Data Preprocessing

DICOM images were converted into the NIfTI format with the use of the dicm2nii tool (https://github.com/xiangruili/dicm2nii). Subsequently, these images were structured according to the Brain Imaging Data Structure (BIDS) guidelines using BIDS‐Matlab (https://bids‐matlab.readthedocs.io/) and custom scripts. The preprocessing of the data was carried out using the fMRIPrep version 23.1.1, via the fmriprep‐docker wrapper (Esteban et al. 2019), a Nipype (RRID: SCR_002502) based tool (Gorgolewski et al. 2011), with the default processing steps.

For each participant, a T1w image underwent correction for intensity nonuniformity using the N4BiasFieldCorrection distributed with antsApplyTransforms (ANTs) (RRID: SCR_004757). Following correction, the T1w images were skull‐stripped employing the antsBrainExtraction workflow, with the OASIS30ANTs serving as the target template. Segmentation of brain tissue into cerebrospinal fluid (CSF), white matter (WM), and gray matter (GM) was conducted on the skull‐stripped T1w image using the FSL fast tool (RRID: SCR_002823). For spatial normalization, the images were aligned to a standard space (MNI152NLin2009cAsym) through nonlinear registration using antsRegistration, which used brain‐extracted versions of the T1w reference and the T1w template.

For each participant, four functional sessions were preprocessed with the following preprocessing steps. Initially, a reference volume and its corresponding skull‐stripped variant were created using a specialized fMRIPrep methodology. Before any spatiotemporal filtering, head‐motion parameters relative to the BOLD reference (including transformation matrices and six rotation and translation parameters) were calculated using mcflirt. The BOLD sequences were then adjusted for slice‐timing using 3dTshift from AFNI (RRID: SCR_005927). Subsequently, the BOLD reference was aligned with the T1w reference via mri_coreg from FreeSurfer and further refined with flirt, employing a boundary‐based registration cost function. This co‐registration was set to six degrees of freedom. Several confounding time series were derived from the preprocessed BOLD data, including framewise displacement (FD), DVARS, and three global signals from the CSF, WM, and the entire brain. In addition, physiological regressors for component‐based noise correction (CompCor) were extracted. Principal components were identified after applying a high‐pass filter (using a discrete cosine filter with a 128‐s cutoff) to the BOLD time series for both temporal (tCompCor) and anatomical (aCompCor) variants of CompCor. The confounding time series from head motion estimates and global signals were enhanced by adding temporal derivatives and quadratic terms. Frames surpassing a 0.5 mm FD or 1.5 standardized DVARS thresholds were marked as motion outliers. Additional nuisance time series were generated through principal component analysis of signals from a narrow band of voxels at the brain's edge. The BOLD time series were then transformed into standard space, producing a preprocessed BOLD run in MNI152NLin2009cAsym space. This transformation used ANTs with Lanczos interpolation to reduce the smoothing effects typically seen with other kernels.

#### 4.3.3 Univariate Activation Analyses

We performed whole‐brain univariate activation analyses using the general linear model (GLM), employing a two‐level approach as implemented in SPM12. At the individual participant level, our design matrix included 14 regressors of interest: “face‐angry,” “face‐disgusted,” “face‐fearful,” “face‐happy,” “face‐sad,” “face‐surprised,” “face‐neutral,” alongside “voice‐angry,” “voice‐disgusted,” “voice‐fearful,” “voice‐happy,” “voice‐sad,” “voice‐surprised,” and “voice‐neutral.” Trials with duration of 12 s were modeled as boxcar functions convolved with the canonical hemodynamic response function (HRF). To account for artifacts, we incorporated the following nuisance regressors: 24 motion‐related regressors (comprising 6 foundational motion parameters, their 6 temporal derivatives, and 12 quadratic terms), the top 5 aCompCor components that explain the most physiological noise variance, the global signal, and the scrubbing regressors (FD > 0.5 mm or DVARS > 1.5). A high‐pass filter with a 128‐s cutoff was applied to filter out low‐frequency noise. Temporal autocorrelation was accounted for with a first‐order autoregressive model AR (1). For each condition and participant, contrast images (i.e., contrast of the average beta‐images across all runs) were generated. These images were subsequently submitted to a second‐level group analysis, treating participants as a random factor. At the group level, we used a flexible factorial design incorporating two within‐subject factors: modality (face, voice) and emotion (angry, disgusted, fearful, happy, sad, surprised, and neutral). Activation maps were thresholded across the whole brain, applying a voxel‐wise significance level of *p* < 0.001, and a cluster‐wise significance level of *p* < 0.05, corrected for family‐wise error (FWE) using Gaussian Random Field theory. To identify regions commonly activated by both facial and vocal expressions, the FWE‐corrected maps for each were analyzed together using a conjunction analysis based on the minimum statistic (Nichols et al. 2005).

#### 4.3.4 Single‐Trial Parameter Estimation

Parameter estimates for each trial were acquired through a “Least Squares Separate” (LSS) method (Mumford et al. 2012; Turner et al. 2012). In this method, a specific trial was modeled with a single regressor, and all the other trials were collapsed into another regressor. Each trial was modeled as a Boxcar regressor convolved with the HRF. A high‐pass filter with a 128‐s cutoff was applied to filter out low‐frequency noise. The GLM also included several nuisance regressors, including 24 motion‐related regressors, the top five components from aCompCor, and the global signal, as previously described. This process was applied iteratively to produce one beta map for each trial, for every participant.

#### 4.3.5 Representational Similarity Analyses

To determine whether brain activations associated with emotional processing reflected individual variability in emotion‐concept knowledge, RSA were performed for faces and voices separately (Kriegeskorte, Mur, and Bandettini 2008). These analyses aimed to determine whether conceptual knowledge between emotions could explain the similarity between corresponding patterns of brain activity. Specifically, RSA tested this within the binary thresholded brain maps associated with processing of facial emotions, vocal emotions, and emotion processing irrespective of facial or vocal modalities. RSA was conducted across each of the three masks using a searchlight analysis approach (Kriegeskorte, Goebel, and Bandettini 2006), implemented in the CoSMoMVPA software package (Oosterhof, Connolly, and Haxby 2016). For each participant, the dissimilarity of activity patterns for different emotions (neural dissimilarity) was compared with the dissimilarity of conceptual knowledge (conceptual dissimilarity) using multiple regression at each 4‐mm radius searchlight sphere, centered on every voxel within any of the three brain masks. Neural dissimilarity was calculated at each searchlight sphere through Pearson distance. This involved correlating brain activity between each pair of emotions (using averaged beta values from single‐trial estimates) across the voxels within a sphere, yielding a symmetrical 7 by 7 matrix. The matrix was subtracted from 1 to generate a dissimilarity matrix. For each searchlight, the lower triangles of both the neural and conceptual dissimilarity matrices were converted into vectors and then analyzed using an ordinary least‐squares regression model. Subsequently, the regression coefficients were Fisher‐transformed and mapped back to their corresponding searchlight centers. At the group level, we performed one‐sided *t*‐tests against zero (because of the interest in positive association) across participants. Significance was determined at the *p* < 0.05 level using a sign‐permutation test, which involved creating null distributions from 10,000 bootstrapping iterations coupled with a threshold‐free cluster‐enhancement procedure controlling for multiple comparisons (Smith and Nichols 2009).

In addition, to account for the impact of basic facial and vocal features on the RSA outcomes, we ran an additional analysis by developing models that capture these low‐level features for both faces and voices. For facial data, we extracted features from the C2 layer in the Hierarchical Model and X (HMAX) model (Serre, Wolf, and Poggio 2005). For vocal data, we calculated pitch using an autocorrelation method, employing the librosa library in Python (McFee et al. 2015). We then measured the Pearson distance between emotions using these low‐level feature models, correlating HMAX or pitch features across each emotion pair. This process produced a symmetrical 7 × 7 matrix. We included these matrices of conceptual and low‐level feature dissimilarity in a multiple regression model to predict neural dissimilarity within each searchlight. This method allowed us to identify brain patterns linked to conceptual knowledge while controlling for the influence of low‐level features. Significance testing was carried out as previously described.

#### 4.3.6 DCM Analyses

We conducted DCM analyses for faces and voices separately to investigate the direction of information flow among brain regions associated with emotion processing (Friston, Harrison, and Penny 2003). First, we constructed a DCM‐specific design matrix, which included the following conditions of interest: “emotion,” “angry,” “disgusted,” “fearful,” “happy,” “sad,” and “surprised.” The “emotion” condition included all trials. Neural responses to “neutral” stimuli were excluded from the model, thereby serving as an implicit baseline. A high‐pass filter with a 128‐s cutoff was applied to filter out low‐frequency noise. Note that we concatenated all sessions of functional data per participant and included a nuisance regressor for sessions to account for the effects of different sessions. Other nuisance regressors included 24 motion‐related regressors, the top five components from aCompCor, and the global signal.

For facial emotion processing, we defined the regions of interest (ROI) based on the results of searchlight RSA for the effects of conceptual knowledge on facial emotion processing. The ROIs included the G_Occipital_Mid‐2‐L [−46 to 73 5], G_Occipital_Inf‐2‐R [51–68 4], and S_Sup_Temporal‐3‐R [46–35 6]. For vocal emotion processing, we defined the ROI based on the results of searchlight RSA for the effects of conceptual knowledge on vocal emotion processing. The ROIs included the G_Temporal_Sup‐4‐L [−64 to 35 5], G_Temporal_Sup‐4‐R [61–27 1], and S_Sup_Temporal‐3‐R [50–36 0]. For each ROI, a sphere with a radius of 10 mm, centered at the group coordinate, served as the basis for searching an individual peak coordinate for each participant within this predefined sphere. This method accounted for variations in the participant‐specific peaks. Subsequently, we created a participant‐specific ROI using a 6 mm radius sphere centered on the identified peak. From this personalized spherical ROI, we extracted the first principal component of significant voxels that passed a threshold of uncorrected *p* < 0.05. The extracted eigenvariate timeseries were adjusted by an *F*‐contrast spanning across all experimental conditions. One participant was excluded from the DCM analyses due to the absence of significant voxels in one of the ROIs.

All DCM analyses were conducted using a two‐level approach with the Parametric Empirical Bayes (PEB) framework implemented in SPM12 (Zeidman, Jafarian, Seghier, et al. 2019). At the first level, we specified and estimated a full model for each participant. This model incorporated all possible bidirectional connections between the three ROIs, including their self‐connections. We set a driving input for each ROI (C matrix). We set the parametric modulator (different emotions) to modulate both the between‐region connections and the self‐connections (B matrix). The neutral emotion was not included in the model, serving as an implicit baseline. The DCM inputs were mean‐centered, so that the A parameters represented the average connectivity across six emotions, compared with the neutral emotion as the baseline. We used variational Laplace for model estimation, which allowed us to acquire posterior estimates of the DCM parameters.

At the second level, we submitted the DCM parameters from each participant to a Bayesian general linear model. Subsequently, we employed Bayesian model reduction (BMR) with an automatic search approach to compare the full model against all nested models in the model space that had different combinations of parameters switched off (Friston et al. 2016). BMR eliminated parameters that did not contribute to the model evidence (as indicated by negative variational free energy), while retaining those parameters with the most evidence. We then performed the Bayesian model averaging (BMA) to average the parameters across models weighted by the evidence supporting each model (Penny et al. 2010). We retained only those parameters for which the posterior probability (Pp) of presence versus absence exceeded 95%.

## 5 Results

### 5.1 Experiment 1

#### 5.1.1 Conceptual Knowledge Varied Across Individuals

**FIGURE 2:**
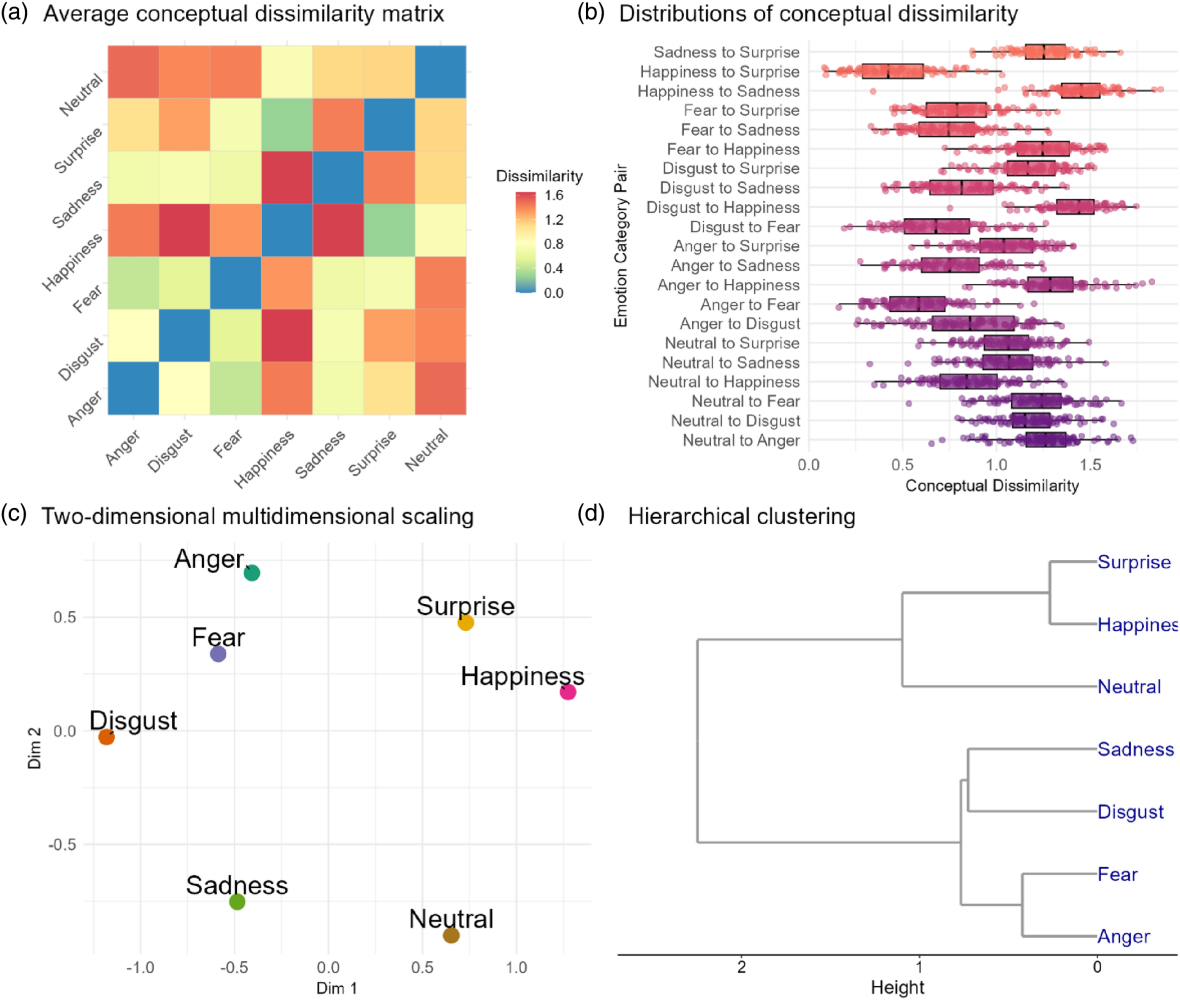
Experiment 1 results. (a) Average conceptual dissimilarity matrix. (b) Distributions of conceptual dissimilarity. (c) Multidimensional scaling results. (d) Hierarchical clustering results.

An inspection of the distributions of conceptual dissimilarity across participants in emotion category pairs revealed substantial inter‐subject variability (Figure 2b). This pronounced variability necessitated the application of personalized conceptual knowledge to pinpoint related brain representations.

#### 5.1.2 MDS Revealed That Valence and Arousal Are Lower‐Dimensional Representations of Emotions

Kruskal's nonmetric MDS revealed a lower‐dimensional framework of emotional conceptual knowledge (Figure 2c). The first dimension reflected valence, ranging from negative emotions (anger, fear, disgust, and sadness) through neutral emotions (surprise and neutral) to positive emotions (happiness). The second dimension reflected arousal, ranging from low arousal emotions (neutral, sadness, and disgust) to high arousal emotions (happiness, anger, fear, and surprise).

#### 5.1.3 Hierarchical Clustering Analysis Showed Meaningful Clusters of Emotions

Hierarchical clustering analysis delineated several clusters of emotions. The cluster included negative emotions with Group 1 comprising fear and anger, Group 2 consisting of sadness and disgust; neutral emotion, encapsulated by neutral itself; and positive emotions, which include happiness and surprise (Figure 2d).

### 5.2 Experiment 2

#### 5.2.1 Behavioral Results

##### 5.2.1.1 Conceptual Knowledge Was Related to Emotion Categorization

**FIGURE 3:**
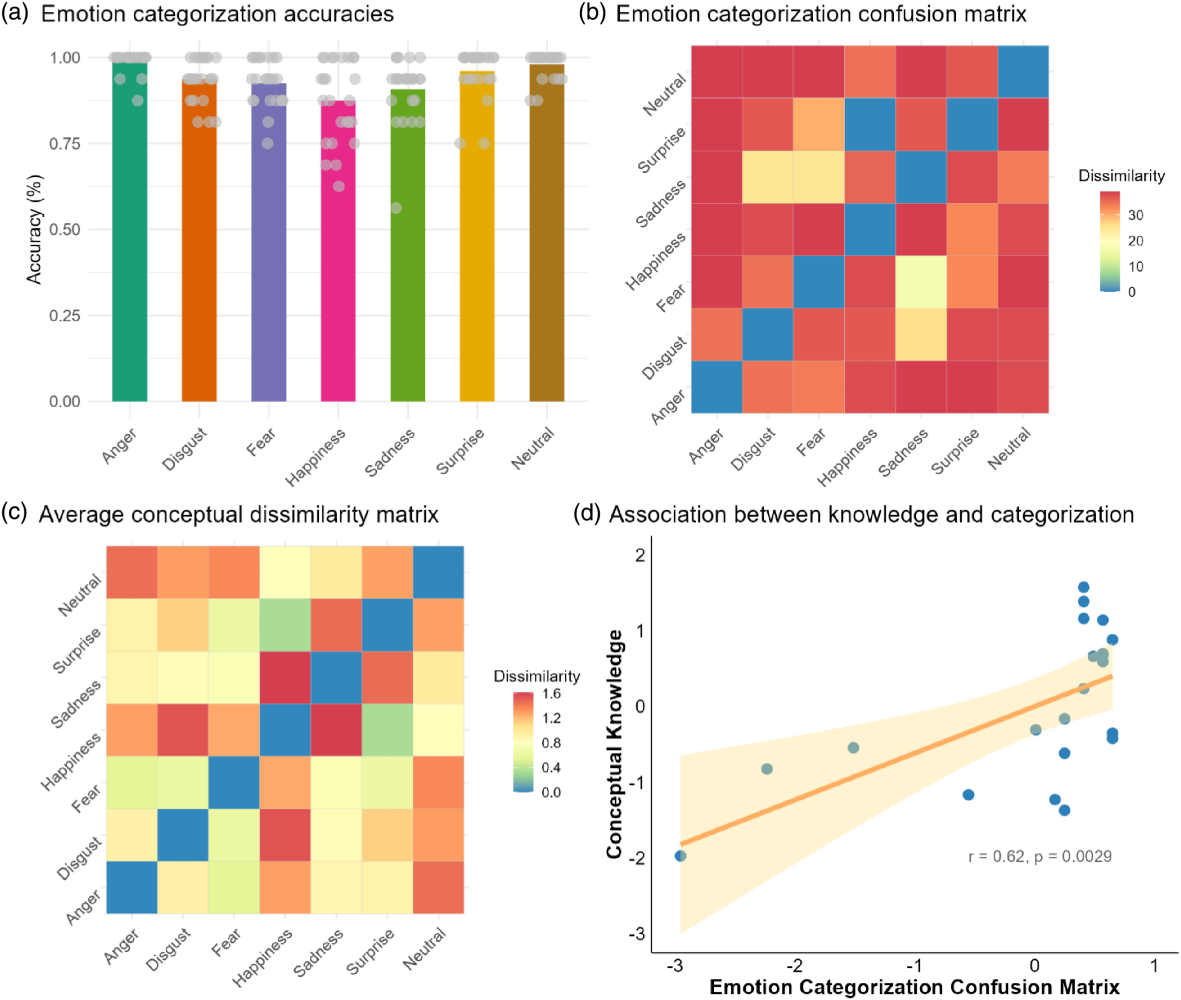
Conceptual knowledge was related to emotion categorization. (a) Average emotion categorization accuracies with individual participant data overlaid. (b) Overall emotion categorization confusion matrix. (c) Average pre‐scan conceptual dissimilarity matrix. (d) Significant correlation between the average conceptual dissimilarity and the overall emotion categorization performances.

The accuracies of emotion categorization were consistently high, as shown in Figure 3a. A significant correlation was observed between the average conceptual dissimilarity and the overall performance in emotion categorization, *r* (19) = 0.62, *p* = 0.0029^1^ (Figure 3b–d). These findings indicate a significant relationship between conceptual knowledge and the ability to categorize emotions behaviorally.

##### 5.2.1.2 Lower‐Dimensional Representation of Conceptual Knowledge Was Comparable to Explicit Valence and Arousal Ratings

**FIGURE 4:**
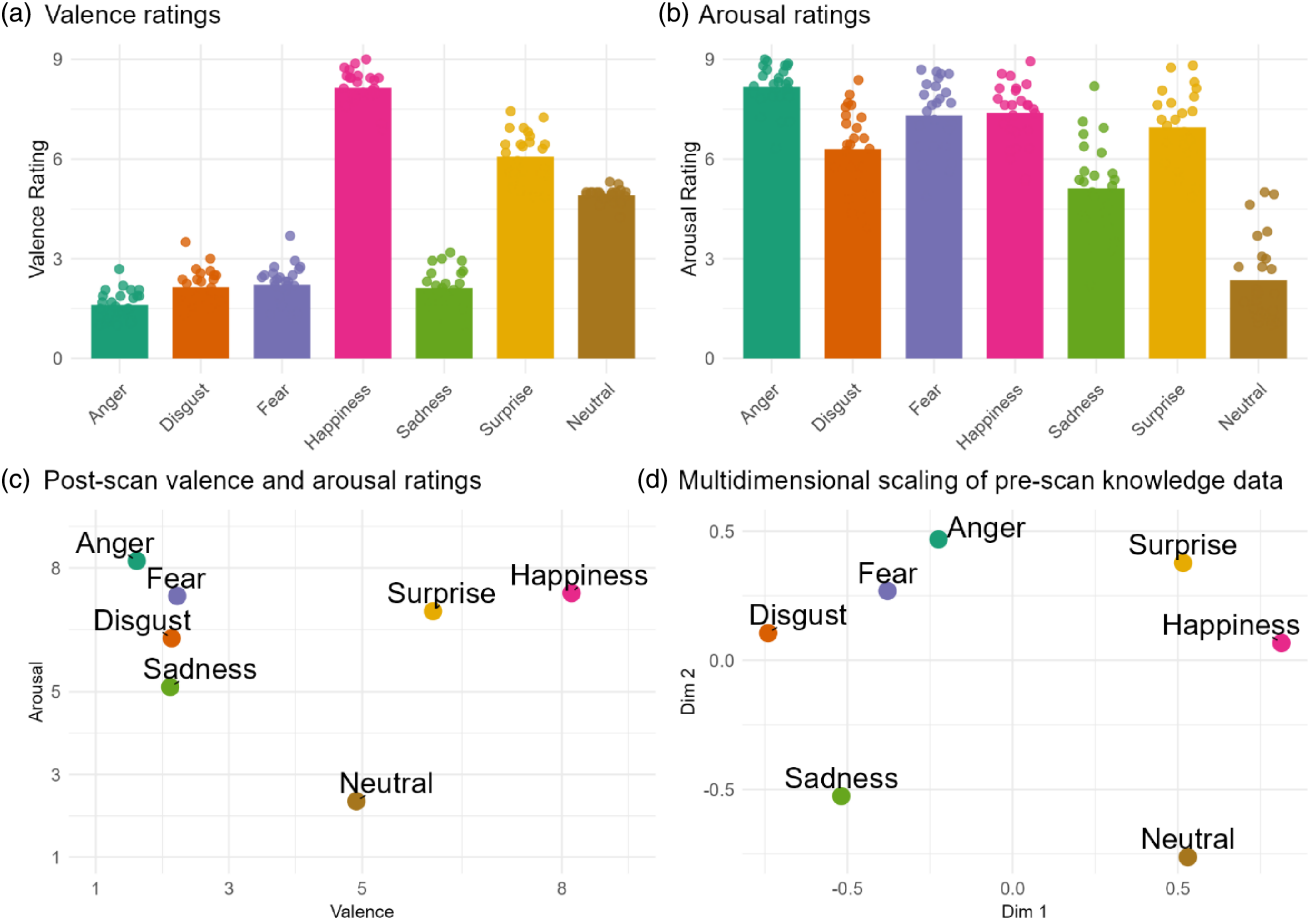
Lower‐dimensional representation of conceptual knowledge was comparable to explicit valence and arousal ratings. (a) Average valence ratings with overlaid individual participant data. (b) Average arousal ratings with individual participant data overlaid. (c) Post‐scan explicit valence and arousal ratings of facial and vocal expressions. (d) Lower‐dimensional representation of pre‐scan conceptual knowledge ratings revealed by multidimensional scaling.

Kruskal's nonmetric MDS revealed a lower‐dimensional structure of emotional conceptual knowledge that aligned with the core‐affect dimensions of valence and arousal. Post‐scan valence and arousal ratings for the same set of facial and vocal expressions also revealed a valence‐arousal circumplex (Figure 4a–d). These results suggest that the primary dimensions underlying conceptual knowledge of emotions are consistent with the valence and arousal framework. Nonetheless, it is important to acknowledge that alternative explanations for these conceptual dimensions cannot be excluded.

### 5.3 fMRI Results

#### 5.3.1 Univariate Activation Results

##### 5.3.1.1 Brain Activations Associated With Facial Emotion Processing

**FIGURE 5:**
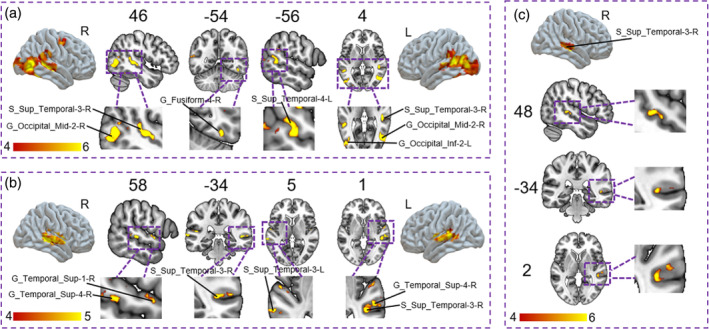
Univariate activation results. (a) Brain activations associated with facial emotion processing. (b) Brain activations associated with vocal emotion processing. (c) Brain activations associated with emotion processing independent of face and voice modality.

We were interested in brain activations during facial emotion processing. Mass‐univariate analysis for the main effect of facial emotion revealed brain activity in the occipital, temporal and precentral brain areas (Table S2, Figure 5a).

##### 5.3.1.2 Brain Activations Associated With Vocal Emotion Processing

We were also interested in brain activations during the vocal emotion processing. Mass‐univariate analysis for the main effect of vocal emotion revealed brain activity in the superior temporal brain regions (Table S3, Figure 5b).

##### 5.3.1.3 Brain Activations Associated With Emotion Processing Independent of Face and Voice Modality

We were also interested in brain activations sensitive to emotion processing, irrespective of its stimulus modality. The conjunction analysis revealed activity in the STS [S_Sup_Temporal‐3‐R, Cluster size = 95, *x* = 48, *y* = −34, *z* = 2, *F*‐value = 8.45] for emotion processing independent of modality (Figure 5c).

#### 5.3.2 RSA Results

##### 5.3.2.1 Brain Representations Associated With Facial Emotion Processing Reflected Individual Variability in Emotion‐Concept Knowledge

**FIGURE 6:**
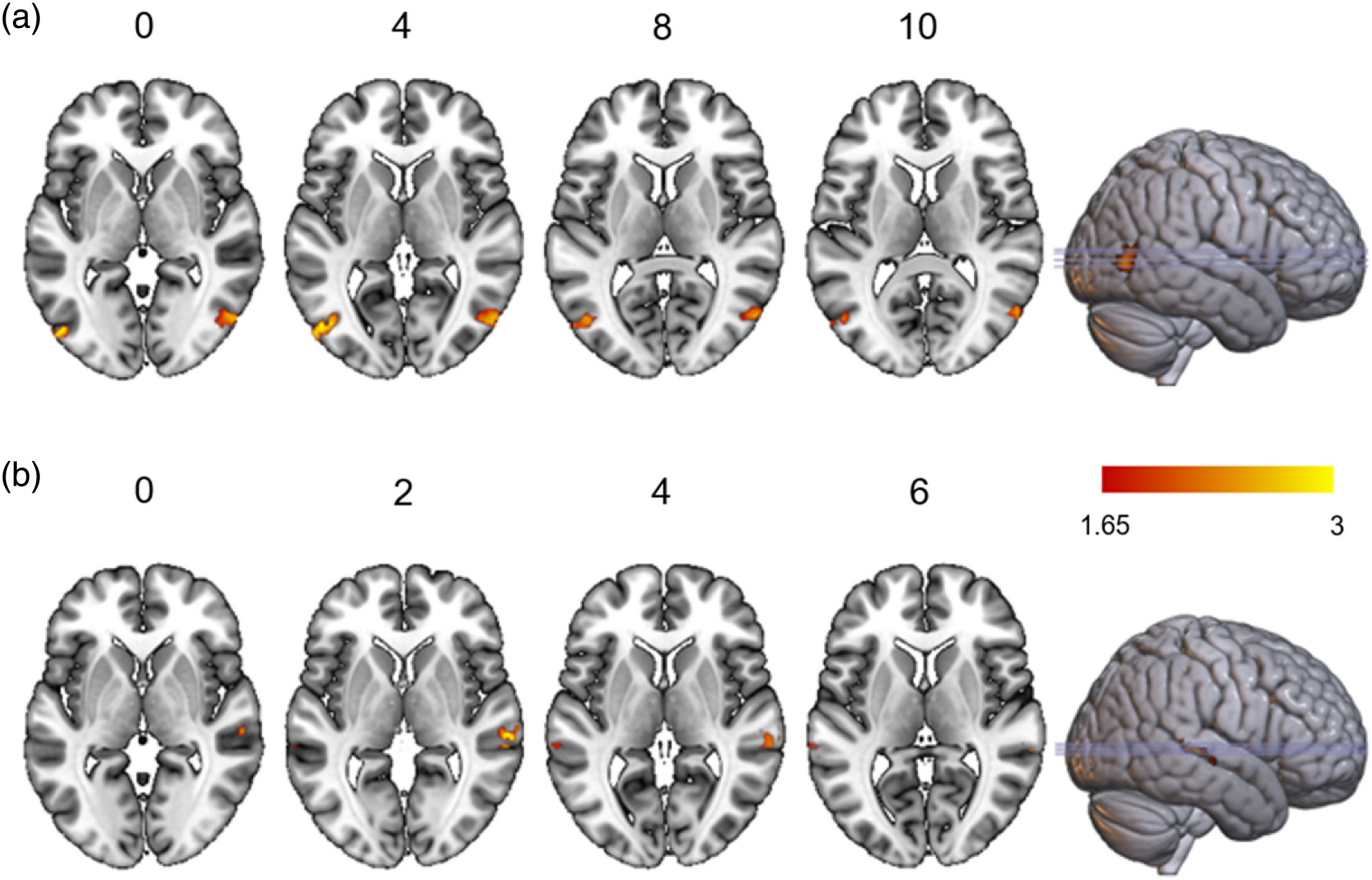
RSA results. (a) Brain representations associated with facial emotion processing reflected individual variability in emotion‐concept knowledge. (b) Brain representations associated with vocal emotion processing reflected individual variability in emotion‐concept knowledge. *Note:* The figure shows threshold‐free cluster‐enhanced *z*‐maps thresholded at a *z*‐score of 1.65, corresponding to *p* < 0.05, one‐tailed (corrected for multiple comparisons).

We aimed to identify the neural patterns that reflected individual variability in emotion‐concept knowledge for facial emotion processing. The searchlight analysis, employing RSA, revealed that the variability in local neural responses was significantly predicted by the variability in conceptual knowledge of emotion in several occipital brain areas (Table 1, Figure 6a). Comparable results were found when controlling for low‐level facial features (Figure S1a).

**TABLE 1:** Results of searchlight RSA for facial emotion processing in the MNI space.

Region	Cluster size	*x*	*y*	*z*
G_Occipital_Lat‐5‐R	2	42	−76	−6
G_Occipital_Mid‐2‐L	181	−46	−73	5
G_Occipital_Inf‐2‐R	200	51	−68	4
G_Occipital_Mid‐3‐R	17	43	−61	13

*Note:* Region names were labeled with the AICHA atlas. Maps were thresholded at the *p* < 0.05 level using a sign‐permutation test, which involved creating null distributions from 10,000 bootstrapping iterations coupled with a threshold‐free cluster‐enhancement procedure controlling for multiple comparisons.

Abbreviations: G = gyrus, L = left hemisphere, R = right hemisphere, S = sulcus.

##### 5.3.2.2 Brain Representations Associated With Vocal Emotion Processing Reflected Individual Variability in Emotion‐Concept Knowledge

We aimed to identify the neural patterns that reflected individual variability in emotion‐concept knowledge for vocal emotion processing. The searchlight analysis, employing RSA, revealed that the variability in local neural responses was significantly predicted by the variability in conceptual knowledge of emotion in superior temporal brain regions (Table 2, Figure 6b). Comparable results were found when controlling for low‐level vocal features (Figure S1b).

**TABLE 2:** Results of searchlight RSA for vocal emotion processing in the MNI space.

Region	Cluster size	*x*	*y*	*z*
G_Temporal_Sup‐4‐R	91	61	−27	1
G_Temporal_Sup‐4‐L	26	−64	−35	5

*Note:* Region names were labeled with the AICHA atlas. Maps were thresholded at the *p* < 0.05 level using a sign‐permutation test, which involved creating null distributions from 10,000 bootstrapping iterations coupled with a threshold‐free cluster‐enhancement procedure controlling for multiple comparisons.

Abbreviations: G = gyrus, L = left hemisphere, R = right hemisphere, S = sulcus.

##### 5.3.2.3 Brain Representations Associated With Emotion Processing Independent of Face and Voice Modality Reflected Individual Variability in Emotion‐Concept Knowledge

We applied searchlight analyses within a 6 mm sphere centered at the peak of the cluster showing significant activation for the conjunction analyses of facial and vocal emotion processing. The searchlight analyses for the face sessions within the modality‐independent region (i.e., right STS) identified a cluster centered at *x* = 46, *y* = −35, *z* = 6 of 12 voxels. The searchlight analyses for the voice sessions within the modality‐independent region (i.e., right STS) identified a cluster centered at *x* = 50, *y* = −36, *z* = 0 of 16 voxels.

#### 5.3.3 DCM Results

##### 5.3.3.1 Emotion‐Independent Connectivity for Facial Emotion Processing

**FIGURE 7:**
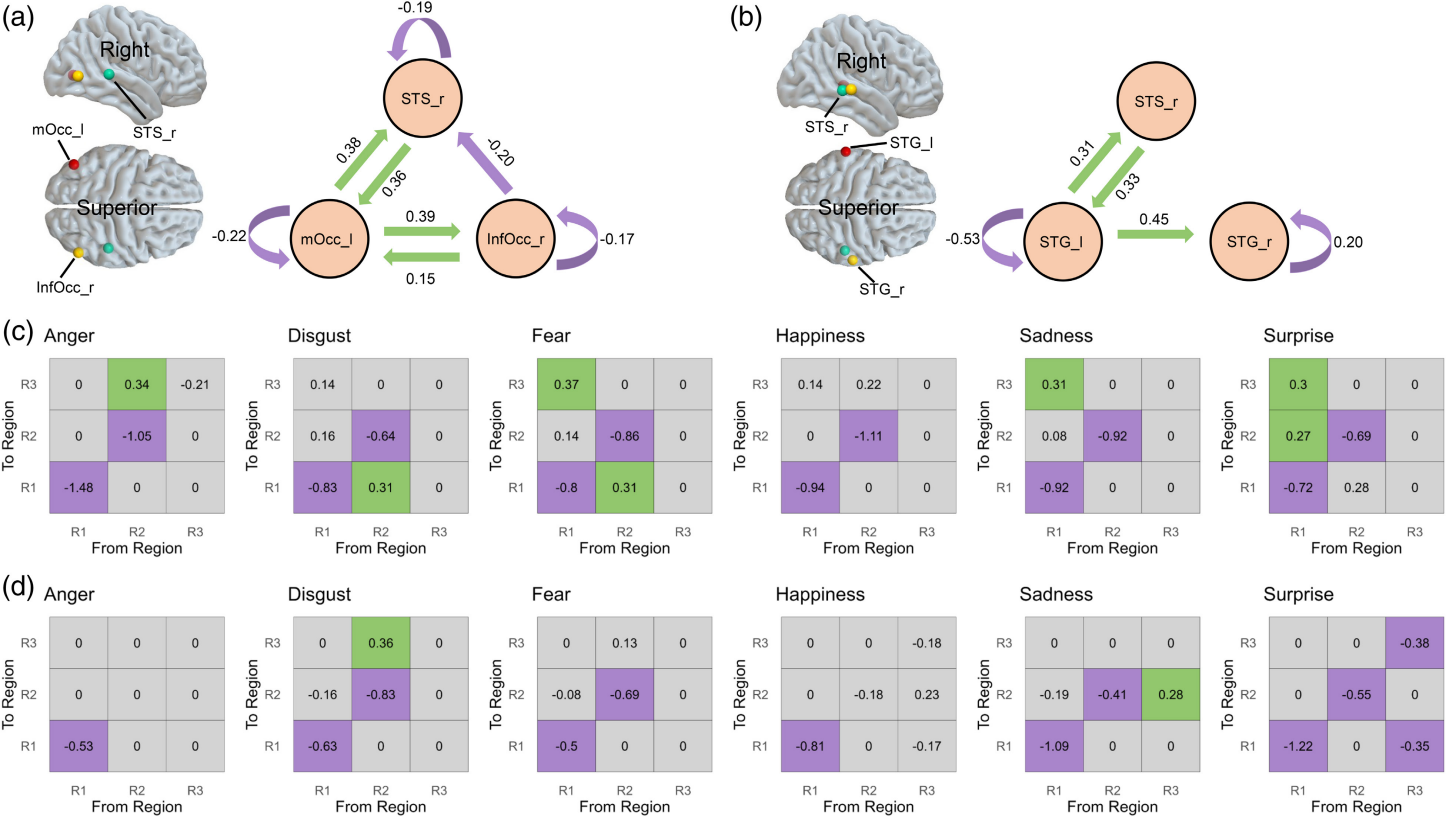
DCM results with values indicating connectivity strength (in Hz). (a) An illustration of three regions and emotion‐independent connectivity between regions for facial emotion processing, thresholded at a posterior probability (Pp) of > 0.95. mOcc_l = left middle occipital cortex; InfOcc_r = right inferior occipital cortex; STS_r = right STS. (b) An illustration of three regions and emotion‐independent connectivity between regions for vocal emotion processing, thresholded at P*p* > 0.95. STG_l = left STG; STG_r = right STG; STS_r = right STS. (c) Emotion‐specific modulatory connectivity between three regions for facial emotion processing. Gray color represents connections with P*p* < 0.95. (d) Emotion‐specific modulatory connectivity between three regions for vocal emotion processing. Gray color represents connections with P*p* < 0.95. Green and purple arrows indicate positive and negative effective connectivity, respectively.

In all three regions implicated for the effects of conceptual knowledge on facial emotion processing, we observed inhibitory self‐connections, which reflected a reduction in the magnitude of the negative self‐connection compared with the neutral condition. In the context of DCM for fMRI, this reduction indicates a release from auto‐inhibition, meaning the region becomes less inhibited and more responsive to inputs from other regions in the network (Zeidman, Jafarian, Corbin, et al. 2019).^2^ There was bidirectional excitatory^3^ connectivity between the left middle occipital cortex and the right STS during facial emotion processing, likely indicating enhanced bottom‐up and top‐down information flows. Similarly, bidirectional excitatory connectivity was observed between the left middle occipital cortex and the right inferior occipital cortex during the processing of facial emotions. Furthermore, we identified an inhibitory connection from the right inferior occipital cortex to the right STS (Figure 7a).

##### 5.3.3.2 Emotion‐Specific Connectivity for Facial Emotion Processing

We observed enhanced inhibitory effects of the left middle occipital and the right inferior occipital cortices for each specific emotion. Additionally, we identified some enhanced excitatory connections for each emotion. Specifically, the connection from the right inferior occipital cortex to the right STS was notably stronger when subjects watched angry faces. Enhanced connections were also observed from the left middle occipital cortex to the right STS during the viewing of faces expressing fear, sadness, or surprise. These enhanced connections likely indicate an improved bottom‐up flow from lower‐level regions to higher‐level regions within the hierarchical pathway. However, we did not observe significant emotion‐specific top‐down modulation effects (Figure 7c).

##### 5.3.3.3 Emotion‐Independent Connectivity for Vocal Emotion Processing

We observed inhibitory self‐connections for two regions, the bilateral superior temporal gyri (STG), but we did not find significant self‐connections for the right STS. There was bidirectional excitatory connectivity between the left STG and the right STS, which likely indicates enhanced bottom‐up and top‐down information flows. Furthermore, we found an excitatory connection from the left STG to the right STG (Figure 7b).

##### 5.3.3.4 Emotion‐Specific Connectivity for Vocal Emotion Processing

We observed enhanced inhibitory effects of the left STG for each specific emotion. We also found enhanced inhibitory effects of the right STG during the viewing of voices expressing disgust, fear, sadness, and surprise, as well as an enhanced inhibitory effect of the right STG for the surprise emotion. Additionally, we found an enhanced connection from the right STG to the right STS for the disgust emotion, and an enhanced connection from the right STS to the right STG. These two effects likely indicate enhanced bottom‐up and top‐down information flows (Figure 7d).

## 6 Discussion

Previous neuroimaging studies have shown that conceptual knowledge of emotions can shape brain representations of facial emotion categories in visual‐perceptual regions (Brooks et al. 2019). However, it remained unclear whether conceptual knowledge influences the representations of emotions in auditory regions for vocal emotions and whether these conceptual effects also manifest at a modality‐independent level across faces and voices. Experiment 1 revealed significant variability in conceptual knowledge among individuals. Experiment 2 demonstrated that individualized conceptual knowledge can predict brain representations in visual regions for faces, auditory regions for voices, and supra‐modal regions across faces and voices. DCM analyses further indicated that the identified modality‐specific and modality‐independent brain regions, which reflect conceptual influences on emotion perception, are interconnected through both bottom‐up and top‐down information flows.

While the effects of conceptual knowledge on emotion perception have been demonstrated (Brooks and Freeman 2018), it was still uncertain at which processing level these effects occur. Brooks et al. (2019) provided evidence that conceptual understanding of emotions predicted neural representations in face‐perceptual regions. Considering the significant similarities between face and voice perception (Belin 2017; Belin, Fecteau, and Bedard 2004; Yovel and Belin 2013), it is plausible to anticipate a similar influence on auditory regions for voices. However, this hypothesis has not been tested, and an opposing outcome could also be valid, considering the alternative perspective that facial and vocal emotion processing utilizes distinct neural mechanisms (Schirmer 2018; Schirmer and Adolphs 2017). Here, leveraging univariate and RSA, we provided evidence that brain representations of emotion categories in both visual and auditory regions (i.e., middle and lateral occipital cortices; STG) are influenced by the conceptual understanding of emotions. Comparable results were found when controlling for low‐level facial or vocal features. These findings are consistent with previous evidence that sensory‐perception regions encode emotional content (Ethofer et al. 2013; Gao and Shinkareva 2021; Kragel et al. 2019). We provided new insights into brain representations of emotion by demonstrating that neural patterns in visual and auditory regions can be associated with participants' own unique conceptual knowledge of emotions.

Beyond the effects in sensory‐perceptual regions, we also discovered that conceptual knowledge impacts supra‐modal representations of emotions in the STS. Previous studies indicate the existence of both modality‐specific and modality‐independent brain regions involved in emotion processing (Chikazoe et al. 2014; Gao and Shinkareva 2021; Young, Frühholz, and Schweinberger 2020). The medial prefrontal cortex and STS have been demonstrated to represent discrete emotion categories at an abstract, modality‐independent level (Lettieri et al. 2024; Peelen, Atkinson, and Vuilleumier 2010). In particular, the STS has been shown to play a key role in audiovisual integration (Driver and Noesselt 2008; Gao et al. 2023). There is also evidence that it represents modality‐independent affective information for visual and auditory modalities, yet it has not been shown to represent such information for the visual and olfactory or visual and gustatory modalities (Chikazoe et al. 2014; Dalenberg et al. 2018; Gao, Weber, and Shinkareva 2019). Consequently, it is plausible that the STS has a role as a specific supra‐modal region dedicated to processing emotions in visual and auditory modalities. Our findings showed that brain representations in the STS were significantly related to individualized conceptual structure, suggesting that conceptual knowledge affects emotion perception at both the modality‐specific level and the modality‐independent level.

DCM results revealed how these modality‐specific and modality‐independent regions work together as a network. In all three nodes, including the left middle occipital gyrus, right inferior occipital gyrus, and right STS, we observed inhibitory self‐connections during facial emotion processing. Within the DCM framework, self‐connection is predefined as negative (Yang et al. 2017; Zeidman, Jafarian, Corbin, et al. 2019), where a reduction in self‐connection (suppression) indicates a release from auto‐inhibition (Schott et al. 2023; Zeidman, Jafarian, Corbin, et al. 2019). This decreased self‐connectivity could signify responses to emotional stimuli compared to neutral stimuli. We observed emotion‐dependent modulation of self‐connections in the left middle occipital and right inferior occipital cortices, but not in the modality‐independent STS. This absence of modulation may suggest that the STS is involved in higher‐level processing. We also found bidirectional excitatory connectivity between the left middle occipital cortex and the right STS, likely indicative of enhanced bottom‐up and top‐down information flows. The bottom‐up connections were further up‐regulated during the viewing of fearful, sad, and surprised faces. Additionally, we found an inhibitory connection from the right inferior occipital cortex to the right STS, which was upregulated during the viewing of angry faces. These results suggest a feed‐forward processing mechanism from the occipital regions to the STS and a feed‐backward mechanism from the STS to the occipital regions. These findings align with previous research that demonstrated enhanced effective connectivity between the right posterior STS, serving as an integration center, and associative auditory and visual cortices like the left middle occipital gyrus and right STG during audiovisual emotional processing (Kreifelts et al. 2007).

For vocal emotion processing, we observed inhibitory self‐connections in the bilateral STG, but not in the right STS. We also noted up‐regulated inhibitory effects in the right STG when processing voices expressing disgust, fear, sadness, and surprise, along with an enhanced inhibitory effect in the right STS specifically for the surprise emotion. Similar to facial expressions, the reduction in self‐connection suggests a release from auto‐inhibition (Schott et al. 2023; Yang et al. 2017; Zeidman, Jafarian, Corbin, et al. 2019). The absence of modulation in the modality‐independent STS may suggest its role in higher‐level processing. There was bidirectional excitatory connectivity between the left STG and the right STS. Additionally, we observed an up‐regulated connection from the right STG to the right STS for the disgust emotion and an enhanced connection from the right STS to the right STG for the surprise emotion. Similar to observations with facial expressions, these findings likely reflect enhanced bottom‐up and top‐down information flows (Kreifelts et al. 2007). Overall, the DCM results provided evidence for both a bottom‐up flow from the modality‐specific regions to the modality‐independent region and a top‐down flow from the modality‐independent region to the modality‐specific regions. The STS, known for its role in processing high‐level socially relevant information, serves as a key node in a hierarchically organized third visual pathway specialized for social processing (Pitcher and Ungerleider 2021). The enhanced bidirectional connections observed between early visual and auditory regions, such as the middle occipital cortex, and the STS suggest a progression toward increasingly abstract representations of emotional content and prioritized communicative interactions between regions when processing emotionally significant stimuli (McMahon, Bonner, and Isik 2023). Moreover, the enhanced connectivity could be interpreted within the framework of predictive coding theory (see Barrett 2017 for the theory of constructed emotion, which aligns with predictive coding). According to this theory, the brain continuously generates predictions about incoming sensory information based on prior experiences and conceptual knowledge. When there is a mismatch between expected and actual input—such as when encountering unexpected emotional stimuli—this prediction error necessitates additional processing to resolve the discrepancy (Friston and Kiebel 2009; Rao and Ballard 1999). The increased bidirectional connectivity between regions, such as the occipital cortex and the STS, may indicate the brain's effort to resolve this prediction error by refining perceptual predictions and updating its interpretation of the sensory environment.

One limitation of our study is that it focused only on a narrow range of emotions, featuring expressions that are unambiguous and extreme, potentially differing from the more nuanced emotional expressions encountered in real life. Research has shown that there are over 20 distinct categories of emotions that manifest in everyday life through facial, vocal, and bodily expressions (Cowen et al. 2018; Cowen and Keltner 2017, 2020). Including a wider range of emotions may reveal richer conceptualizations and a broader array of dimensions. Nonetheless, as demonstrated in Experiment 1, we observed significant variability in conceptual knowledge across individuals. The influence of conceptual knowledge on these unambiguous expressions underscores the robustness of these effects (Brooks et al. 2019; Brooks and Freeman 2018). Future research should delve deeper into whether the effects of conceptual knowledge on emotion perception apply to other relatively ambiguous emotion categories and investigate the underlying neural mechanisms involved.

## Ethics Statement

This study was approved by the University of South Carolina Institutional Review Board.

## Conflicts of Interest

The authors declare no conflicts of interest.

## Supporting information


Data S1.

## Data Availability

The data that support the findings of this study are available on request from the corresponding author. The data are not publicly available due to privacy or ethical restrictions.

## Endnotes

^1^
We computed Cook's distance, a measure of the overall influence of a case on the model, to identify any influential cases. The maximum value was equal to 0.141, which is below the threshold of 1, indicating that there were no extreme points in this model for fitting the sampled data.

^2^
Self‐connections are modeled as negative in DCM to ensure neural activity remains stable, preventing “run‐away excitation” within the network. This negative self‐connection represents an inhibitory mechanism within each brain region, balancing excitatory inputs and maintaining the stability of neural dynamics.

^3^
In the context of DCM for fMRI, the term “excitatory” is used to describe a positive influence, indicating that an increase in activity in one region leads to an increase in activity in another. However, it is important to note that this term reflects a model‐based inference from BOLD signal data rather than a direct measurement of cellular‐level excitatory processes.

## References

[hbm70040-bib-0001] Barrett, L. F. 2014. “The Conceptual Act Theory: A précis.” Emotion Review 6, no. 4: 292–297.

[hbm70040-bib-0002] Barrett, L. F. 2017. “The Theory of Constructed Emotion: An Active Inference Account of Interoception and Categorization.” Social Cognitive and Affective Neuroscience 12, no. 1: 1–23. 27798257 10.1093/scan/nsw154 PMC5390700

[hbm70040-bib-0003] Barrett, L. F. , and K. A. Lindquist . 2008. “The Embodiment of Emotion.” In Embodied Grounding: Social, Cognitive, Affective, and Neuroscientific Approaches, 237–262. New York: Cambridge University Press.

[hbm70040-bib-0004] Belin, P. 2017. “Similarities in Face and Voice Cerebral Processing.” Visual Cognition 25, no. 4–6: 658–665.

[hbm70040-bib-0005] Belin, P. , S. Fecteau , and C. Bedard . 2004. “Thinking the Voice: Neural Correlates of Voice Perception.” Trends in Cognitive Sciences 8, no. 3: 129–135. 15301753 10.1016/j.tics.2004.01.008

[hbm70040-bib-0006] Boersma, P. , and D. Weenink . 2009. PRAAT: Doing Phonetics by Computer (Version 5.1. 05) [computer Program].

[hbm70040-bib-0007] Brooks, J. A. , J. Chikazoe , N. Sadato , and J. B. Freeman . 2019. “The Neural Representation of Facial‐Emotion Categories Reflects Conceptual Structure.” Proceedings of the National Academy of Sciences 116, no. 32: 15861–15870. 10.1073/pnas.1816408116 PMC6689944 31332015

[hbm70040-bib-0008] Brooks, J. A. , and J. B. Freeman . 2018. “Conceptual Knowledge Predicts the Representational Structure of Facial Emotion Perception.” Nature Human Behaviour 2, no. 8: 581–591. https://www.nature.com/articles/s41562‐018‐0376‐6.pdf. 10.1038/s41562-018-0376-6 PMC6788630 31209318

[hbm70040-bib-0009] Brück, C. , B. Kreifelts , and D. Wildgruber . 2011. “Emotional Voices in Context: A Neurobiological Model of Multimodal Affective Information Processing.” Physics of Life Reviews 8, no. 4: 383–403. 22035772 10.1016/j.plrev.2011.10.002

[hbm70040-bib-0010] Chikazoe, J. , D. H. Lee , N. Kriegeskorte , and A. K. Anderson . 2014. “Population Coding of Affect Across Stimuli, Modalities and Individuals.” Nature Neuroscience 17, no. 8: 1114–1122. 24952643 10.1038/nn.3749 PMC4317366

[hbm70040-bib-0011] Cowen, A. S. , H. A. Elfenbein , P. Laukka , and D. Keltner . 2018. “Mapping 24 Emotions Conveyed by Brief Human Vocalization.” American Psychologist 74, no. 6: 698. 30570267 10.1037/amp0000399 PMC6586540

[hbm70040-bib-0012] Cowen, A. S. , and D. Keltner . 2017. “Self‐Report Captures 27 Distinct Categories of Emotion Bridged by Continuous Gradients.” Proceedings of the National Academy of Sciences 114, no. 38: E7900–E7909. 10.1073/pnas.1702247114 PMC5617253 28874542

[hbm70040-bib-0013] Cowen, A. S. , and D. Keltner . 2020. “What the Face Displays: Mapping 28 Emotions Conveyed by Naturalistic Expression.” American Psychologist 75, no. 3: 349–364. 31204816 10.1037/amp0000488 PMC6917997

[hbm70040-bib-0014] Cox, T. F. , and M. A. Cox . 2000. Multidimensional scaling. Boca Raton, Florida: CRC Press.

[hbm70040-bib-0015] Dalenberg, J. R. , L. Weitkamp , R. J. Renken , and G. J. ter Horst . 2018. “Valence Processing Differs Across Stimulus Modalities.” NeuroImage 183: 734–744. 30165252 10.1016/j.neuroimage.2018.08.059

[hbm70040-bib-0016] Driver, J. , and T. Noesselt . 2008. “Multisensory Interplay Reveals Crossmodal Influences on ‘Sensory‐specific’ brain Regions, Neural Responses, and Judgments.” Neuron 57, no. 1: 11–23. 18184561 10.1016/j.neuron.2007.12.013 PMC2427054

[hbm70040-bib-0017] Ekman, P. , and D. Cordaro . 2011. “What Is Meant by Calling Emotions Basic.” Emotion Review 3, no. 4: 364–370.

[hbm70040-bib-0018] Esteban, O. , C. J. Markiewicz , R. W. Blair , et al. 2019. “fMRIPrep: A Robust Preprocessing Pipeline for Functional MRI.” Nature Methods 16, no. 1: 111–116. 30532080 10.1038/s41592-018-0235-4 PMC6319393

[hbm70040-bib-0019] Ethofer, T. , J. Bretscher , S. Wiethoff , et al. 2013. “Functional Responses and Structural Connections of Cortical Areas for Processing Faces and Voices in the Superior Temporal Sulcus.” NeuroImage 76: 45–56. 23507387 10.1016/j.neuroimage.2013.02.064

[hbm70040-bib-0020] Freeman, J. B. , and N. Ambady . 2011. “A Dynamic Interactive Theory of Person Construal.” Psychological Review 118, no. 2: 247–279. 21355661 10.1037/a0022327

[hbm70040-bib-0021] Freeman, J. B. , R. M. Stolier , and J. A. Brooks . 2020. “Dynamic Interactive Theory as a Domain‐General Account of Social Perception.” In Advances in Experimental Social Psychology, vol. 61, 237–287. Amsterdam, Netherlands: Elsevier. 34326560 10.1016/bs.aesp.2019.09.005 PMC8317542

[hbm70040-bib-0022] Friston, K. , and S. Kiebel . 2009. “Predictive Coding Under the Free‐Energy Principle.” Philosophical Transactions of the Royal Society, B: Biological Sciences 364, no. 1521: 1211–1221. 10.1098/rstb.2008.0300 PMC2666703 19528002

[hbm70040-bib-0023] Friston, K. J. , L. Harrison , and W. Penny . 2003. “Dynamic causal modelling.” NeuroImage 19, no. 4: 1273–1302. 12948688 10.1016/s1053-8119(03)00202-7

[hbm70040-bib-0024] Friston, K. J. , V. Litvak , A. Oswal , et al. 2016. “Bayesian Model Reduction and Empirical Bayes for Group (DCM) Studies.” NeuroImage 128: 413–431. 26569570 10.1016/j.neuroimage.2015.11.015 PMC4767224

[hbm70040-bib-0025] Gao, C. , J. J. Green , X. Yang , S. Oh , J. Kim , and S. V. Shinkareva . 2023. “Audiovisual Integration in the Human Brain: A Coordinate‐Based Meta‐Analysis.” Cerebral Cortex 33, no. 9: 5574–5584. 36336347 10.1093/cercor/bhac443 PMC10152097

[hbm70040-bib-0026] Gao, C. , and S. V. Shinkareva . 2021. “Modality‐General and Modality‐Specific Audiovisual Valence Processing.” Cortex 138: 127–137. 33684626 10.1016/j.cortex.2021.01.022

[hbm70040-bib-0027] Gao, C. , C. E. Weber , and S. V. Shinkareva . 2019. “The Brain Basis of Audiovisual Affective Processing: Evidence From a Coordinate‐Based Activation Likelihood Estimation Meta‐Analysis.” Cortex 120: 66–77. 31255920 10.1016/j.cortex.2019.05.016

[hbm70040-bib-0028] Gendron, M. , K. A. Lindquist , L. Barsalou , and L. F. Barrett . 2012. “Emotion Words Shape Emotion Percepts.” Emotion 12, no. 2: 314–325. 22309717 10.1037/a0026007 PMC4445832

[hbm70040-bib-0029] Gorgolewski, K. , C. D. Burns , C. Madison , et al. 2011. “Nipype: A Flexible, Lightweight and Extensible Neuroimaging Data Processing Framework in Python.” Frontiers in Neuroinformatics 5: 13. 21897815 10.3389/fninf.2011.00013 PMC3159964

[hbm70040-bib-0030] Kim, J. , S. V. Shinkareva , and D. H. Wedell . 2017. “Representations of Modality‐General Valence for Videos and Music Derived From fMRI Data.” NeuroImage 148: 42–54. 28057489 10.1016/j.neuroimage.2017.01.002

[hbm70040-bib-0031] Kragel, P. A. , M. C. Reddan , K. S. LaBar , and T. D. Wager . 2019. “Emotion Schemas Are Embedded in the Human Visual System.” Science Advances 5, no. 7: eaaw4358. 10.1126/sciadv.aaw4358. 31355334 PMC6656543

[hbm70040-bib-0032] Kreifelts, B. , T. Ethofer , W. Grodd , M. Erb , and D. Wildgruber . 2007. “Audiovisual Integration of Emotional Signals in Voice and Face: An Event‐Related fMRI Study.” NeuroImage 37, no. 4: 1445–1456. 17659885 10.1016/j.neuroimage.2007.06.020

[hbm70040-bib-0033] Kriegeskorte, N. , R. Goebel , and P. Bandettini . 2006. “Information‐Based Functional Brain Mapping.” Proceedings of the National Academy of Sciences 103, no. 10: 3863–3868. 10.1073/pnas.0600244103 PMC1383651 16537458

[hbm70040-bib-0034] Kriegeskorte, N. , M. Mur , and P. A. Bandettini . 2008. “Representational Similarity Analysis‐Connecting the Branches of Systems Neuroscience.” Frontiers in Systems Neuroscience 2: 249. 10.3389/neuro.06.004.2008 PMC2605405 19104670

[hbm70040-bib-0035] Lettieri, G. , G. Handjaras , E. M. Cappello , et al. 2024. “Dissecting Abstract, Modality‐Specific and Experience‐Dependent Coding of Affect in the Human Brain.” Science Advances 10, no. 10: eadk6840. 38457501 10.1126/sciadv.adk6840 PMC10923499

[hbm70040-bib-0036] Lindquist, K. A. 2017. “The Role of Language in Emotion: Existing Evidence and Future Directions.” Current Opinion in Psychology 17: 135–139. 28950959 10.1016/j.copsyc.2017.07.006

[hbm70040-bib-0037] Lindquist, K. A. , L. F. Barrett , E. Bliss‐Moreau , and J. A. Russell . 2006. “Language and the Perception of Emotion.” Emotion 6, no. 1: 125–138. 16637756 10.1037/1528-3542.6.1.125

[hbm70040-bib-0038] Lindquist, K. A. , and M. Gendron . 2013. “What's in a Word? Language Constructs Emotion Perception.” Emotion Review 5, no. 1: 66–71.

[hbm70040-bib-0039] Lindquist, K. A. , A. B. Satpute , and M. Gendron . 2015. “Does Language Do More Than Communicate Emotion?” Current Directions in Psychological Science 24, no. 2: 99–108. 25983400 10.1177/0963721414553440 PMC4428906

[hbm70040-bib-0040] Livingstone, S. R. , and F. A. Russo . 2018. “The Ryerson Audio‐Visual Database of Emotional Speech and Song (RAVDESS): A Dynamic, Multimodal Set of Facial and Vocal Expressions in North American English.” PLoS One 13, no. 5: e0196391. 29768426 10.1371/journal.pone.0196391 PMC5955500

[hbm70040-bib-0041] McFee, B. , C. Raffel , D. Liang , et al. 2015. “Librosa: Audio and Music Signal Analysis in Python.” SciPy 8: 18–25.

[hbm70040-bib-0042] McMahon, E. , M. F. Bonner , and L. Isik . 2023. “Hierarchical Organization of Social Action Features Along the Lateral Visual Pathway.” Current Biology 33, no. 23: 5035–5047. e5038. 37918399 10.1016/j.cub.2023.10.015 PMC10841461

[hbm70040-bib-0043] Mumford, J. A. , B. O. Turner , F. G. Ashby , and R. A. Poldrack . 2012. “Deconvolving BOLD Activation in Event‐Related Designs for Multivoxel Pattern Classification Analyses.” NeuroImage 59, no. 3: 2636–2643. 21924359 10.1016/j.neuroimage.2011.08.076 PMC3251697

[hbm70040-bib-0044] Murtagh, F. , and P. Legendre . 2014. “Ward's Hierarchical Agglomerative Clustering Method: Which Algorithms Implement Ward's Criterion?” Journal of Classification 31, no. 3: 274–295.

[hbm70040-bib-0045] Nichols, T. , M. Brett , J. Andersson , T. Wager , and J.‐B. Poline . 2005. “Valid Conjunction Inference With the Minimum Statistic.” NeuroImage 25, no. 3: 653–660. 15808966 10.1016/j.neuroimage.2004.12.005

[hbm70040-bib-0046] Niedenthal, P. M. 2008. “Emotion Concepts.” In Handbook of Emotions, vol. 3, 587–600. New York: The Guilford Press.

[hbm70040-bib-0047] Nook, E. C. , K. A. Lindquist , and J. Zaki . 2015. “A New Look at Emotion Perception: Concepts Speed and Shape Facial Emotion Recognition.” Emotion 15, no. 5: 569–578. 25938612 10.1037/a0039166

[hbm70040-bib-0048] Oosterhof, N. N. , A. C. Connolly , and J. V. Haxby . 2016. “CoSMoMVPA: Multi‐Modal Multivariate Pattern Analysis of Neuroimaging Data in Matlab/GNU Octave.” Frontiers in Neuroinformatics 10: 27. 27499741 10.3389/fninf.2016.00027 PMC4956688

[hbm70040-bib-0049] Peelen, M. V. , A. P. Atkinson , and P. Vuilleumier . 2010. “Supramodal Representations of Perceived Emotions in the Human Brain.” Journal of Neuroscience 30, no. 30: 10127–10134. 20668196 10.1523/JNEUROSCI.2161-10.2010 PMC6633378

[hbm70040-bib-0050] Penny, W. D. , K. E. Stephan , J. Daunizeau , et al. 2010. “Comparing Families of Dynamic Causal Models.” PLoS Computational Biology 6, no. 3: e1000709. 20300649 10.1371/journal.pcbi.1000709 PMC2837394

[hbm70040-bib-0051] Pitcher, D. , and L. G. Ungerleider . 2021. “Evidence for a Third Visual Pathway Specialized for Social Perception.” Trends in Cognitive Sciences 25, no. 2: 100–110. 33334693 10.1016/j.tics.2020.11.006 PMC7811363

[hbm70040-bib-0052] Rao, R. P. , and D. H. Ballard . 1999. “Predictive Coding in the Visual Cortex: A Functional Interpretation of Some Extra‐Classical Receptive‐Field Effects.” Nature Neuroscience 2, no. 1: 79–87. 10195184 10.1038/4580

[hbm70040-bib-0053] Sabatinelli, D. , E. E. Fortune , Q. Li , et al. 2011. “Emotional Perception: Meta‐Analyses of Face and Natural Scene Processing.” NeuroImage 54, no. 3: 2524–2533. 20951215 10.1016/j.neuroimage.2010.10.011

[hbm70040-bib-0054] Schirmer, A. 2018. “Is the Voice an Auditory Face? An ALE Meta‐Analysis Comparing Vocal and Facial Emotion Processing.” Social Cognitive and Affective Neuroscience 13, no. 1: 1–13. 29186621 10.1093/scan/nsx142 PMC5793823

[hbm70040-bib-0055] Schirmer, A. , and R. Adolphs . 2017. “Emotion Perception From Face, Voice, and Touch: Comparisons and Convergence.” Trends in Cognitive Sciences 21, no. 3: 216–228. 28173998 10.1016/j.tics.2017.01.001 PMC5334135

[hbm70040-bib-0056] Schott, B. H. , J. Soch , J. M. Kizilirmak , et al. 2023. “Inhibitory Temporo‐Parietal Effective Connectivity Is Associated With Explicit Memory Performance in Older Adults.” Iscience 26, no. 10: 107765. 37744028 10.1016/j.isci.2023.107765 PMC10514462

[hbm70040-bib-0057] Serre, T. , L. Wolf , and T. Poggio . 2005. “Object Recognition With Features Inspired by Visual Cortex.” In Computer Vision and Pattern Recognition (CVPR 2005), San Diego, CA, USA: IEEE.

[hbm70040-bib-0058] Shinkareva, S. V. , C. Gao , and D. Wedell . 2020. “Audiovisual Representations of Valence: A Cross‐Study Perspective.” Affective Science 1, no. 4: 237–246. 36042819 10.1007/s42761-020-00023-9 PMC9382970

[hbm70040-bib-0059] Shinkareva, S. V. , J. Wang , and D. H. Wedell . 2013. “Examining Similarity Structure: Multidimensional Scaling and Related Approaches in Neuroimaging.” Computational and Mathematical Methods in Medicine 2013: 796183. 23662162 10.1155/2013/796183 PMC3639644

[hbm70040-bib-0060] Smith, S. M. , and T. E. Nichols . 2009. “Threshold‐Free Cluster Enhancement: Addressing Problems of Smoothing, Threshold Dependence and Localisation in Cluster Inference.” NeuroImage 44, no. 1: 83–98. 18501637 10.1016/j.neuroimage.2008.03.061

[hbm70040-bib-0061] Turner, B. O. , J. A. Mumford , R. A. Poldrack , and F. G. Ashby . 2012. “Spatiotemporal Activity Estimation for Multivoxel Pattern Analysis With Rapid Event‐Related Designs.” NeuroImage 62, no. 3: 1429–1438. 22659443 10.1016/j.neuroimage.2012.05.057 PMC3408801

[hbm70040-bib-0062] van Rijn, P. , and P. Larrouy‐Maestri . 2023. “Modelling Individual and Cross‐Cultural Variation in the Mapping of Emotions to Speech Prosody.” Nature Human Behaviour 7, no. 3: 386–396. 10.1038/s41562-022-01505-5 PMC10038802 36646838

[hbm70040-bib-0063] Yang, Y. , N. Zhong , K. Friston , et al. 2017. “The Functional Architectures of Addition and Subtraction: Network Discovery Using fMRI and DCM.” Human Brain Mapping 38, no. 6: 3210–3225. 28345153 10.1002/hbm.23585 PMC6866939

[hbm70040-bib-0064] Young, A. W. , S. Frühholz , and S. R. Schweinberger . 2020. “Face and Voice Perception: Understanding Commonalities and Differences.” Trends in Cognitive Sciences 24, no. 5: 398–410. 32298625 10.1016/j.tics.2020.02.001

[hbm70040-bib-0065] Yovel, G. , and P. Belin . 2013. “A Unified Coding Strategy for Processing Faces and Voices.” Trends in Cognitive Sciences 17, no. 6: 263–271. 23664703 10.1016/j.tics.2013.04.004 PMC3791405

[hbm70040-bib-0066] Zeidman, P. , A. Jafarian , N. Corbin , et al. 2019. “A Guide to Group Effective Connectivity Analysis, Part 1: First Level Analysis With DCM for fMRI.” NeuroImage 200: 174–190. 31226497 10.1016/j.neuroimage.2019.06.031 PMC6711459

[hbm70040-bib-0067] Zeidman, P. , A. Jafarian , M. L. Seghier , et al. 2019. “A Guide to Group Effective Connectivity Analysis, Part 2: Second Level Analysis With PEB.” NeuroImage 200: 12–25. 31226492 10.1016/j.neuroimage.2019.06.032 PMC6711451

