# Supplementary material for: Neural Representations of Emotions in Visual, Auditory, and Modality‐Independent Regions Reflect Idiosyncratic Conceptual Knowledge
Source: Hum Brain Mapp. 2024 Oct 12;45(14):e70040. doi: 10.1002/hbm.70040 (PMC11470372; doi:10.1002/hbm.70040)
Supplement: Supplementary file 1 — Data S1. [file HBM-45-e70040-s001.docx]

**Supplemental Information**

**Supplemental Table 1. 40 words and phrases.**

| crying | smiling | nausea | yelling | shaking |
| --- | --- | --- | --- | --- |
| shock | heart racing | grossness | frowning | jumping |
| wide eyes | upset | vomiting | laughing | heart |
| excitement | sweating | sickness | loving | depression |
| calm | tense | avoidance | slumping over | screaming |
| clenching fists | lonely | pain | frustration | grasping |
| warmth | hiding | rage | punching | anxious |
| headache | gagging | turning away | lethargic | jaw grinding |

**Supplemental Table 2. Results of mass-univariate analysis for the main effect of facial emotion in the MNI space.**

| Region | Cluster size | x | y | z | F-value |
| --- | --- | --- | --- | --- | --- |
| G_Occipital_Mid-2-R | 513 | 46 | -66 | 6 | 22.26 |
| S_Sup_Temporal-3-R | 283 | 48 | -32 | 4 | 12.70 |
| G_Fusiform-4-R | 72 | 42 | -54 | -14 | 11.70 |
| S_Sup_Temporal-4-L | 569 | -46 | -46 | 10 | 10.24 |
| G_Fusiform-4-L | 48 | -42 | -46 | -12 | 7.93 |
| G_SupraMarginal-2-R | 27 | 62 | -32 | 24 | 7.82 |
| S_Sup_Temporal-4-L | 48 | -56 | -44 | 26 | 7.52 |
| Unknown | 35 | 66 | -46 | 20 | 7.23 |
| G_Occipital_Lat-2-R | 23 | 28 | -90 | 10 | 6.33 |
| G_Temporal_Sup-4-L | 20 | -64 | -28 | 4 | 6.24 |
| G_Occipital_Lat-3-L | 24 | -36 | -92 | -10 | 6.10 |
| G_Occipital_Lat-5-L | 16 | -40 | -78 | -10 | 5.99 |
| G_Occipital_Lat-4-R | 15 | 42 | -84 | 2 | 5.62 |
| S_Precentral-4-R | 27 | 48 | 2 | 54 | 5.60 |
| G_Occipital_Lat-4-L | 33 | -22 | -88 | 8 | 5.58 |
| G_Calcarine-3-R | 13 | 18 | -102 | 4 | 5.53 |

Notes. Region names were labeled with the AICHA atlas. Activation maps were thresholded with cluster-wise FWE correction, *p* < 0.05 (initial voxel-wise threshold *p* < 0.001). L = left hemisphere, R = right hemisphere, S = Sulcus, G = Gyrus.

**Supplemental table 3. Results of mass-univariate analysis for the main effect of vocal emotion in the MNI space.**

| Region | Cluster size | x | y | z | F-value |
| --- | --- | --- | --- | --- | --- |
| G_Temporal_Sup-4-R | 181 | 64 | -18 | -4 | 9.73 |
| G_Temporal_Sup-3-L | 73 | -54 | -8 | 4 | 7.63 |
| G_Temporal_Sup-3-L | 51 | -44 | -34 | 12 | 7.33 |
| G_Temporal_Sup-1-R | 81 | 58 | 0 | 0 | 7.26 |
| G_Temporal_Sup-4-L | 44 | -62 | -34 | 6 | 7.09 |
| Unknown | 11 | -26 | 50 | -34 | 6.14 |
| S_Sup_Temporal-3-L | 17 | -54 | -34 | 4 | 6.06 |
| G_Temporal_Sup-3-R | 17 | 52 | -28 | 12 | 5.56 |
| G_Temporal_Sup-3-L | 17 | -56 | -24 | 12 | 5.56 |
| G_Temporal_Sup-4-L | 15 | -66 | -24 | 6 | 5.30 |

Notes. Region names were labeled with the AICHA atlas. Activation maps were thresholded with cluster-wise FWE correction, *p* < 0.05 (initial voxel-wise threshold *p* < 0.001). L = left hemisphere, R = right hemisphere, S = Sulcus, G = Gyrus.


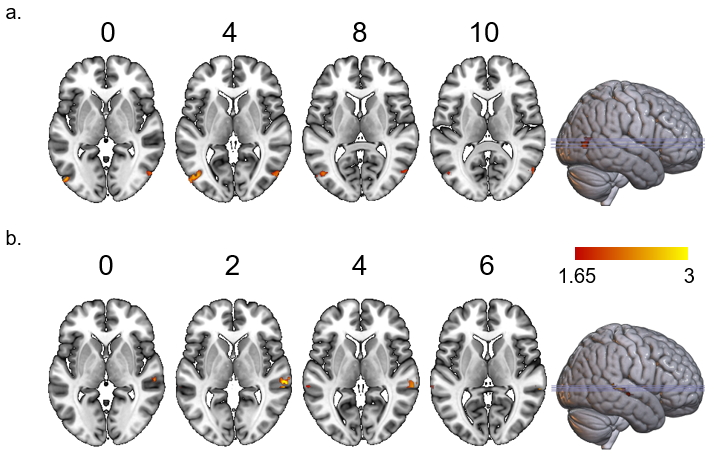


***Supplemental Fig 1. RSA results controlling for low-level features.*** *a) Brain representations associated with facial emotion processing reflected individual variability in emotion-concept knowledge controlling low-level facial features. b) Brain representations associated with vocal emotion processing reflected individual variability in emotion-concept knowledge controlling low-level vocal features*. *Note. The figure shows threshold-free cluster-enhanced z-maps thresholded at a z-score of 1.65, corresponding to p < 0.05, one-tailed (corrected for multiple comparisons).*
